# Conscientious objection to participation in abortion by midwives and nurses: a systematic review of reasons

**DOI:** 10.1186/s12910-018-0268-3

**Published:** 2018-04-27

**Authors:** Valerie Fleming, Lucy Frith, Ans Luyben, Beate Ramsayer

**Affiliations:** 1 Faculty of Education and Health, Liverpool John Moores University, Henry Cotton Building, Trueman Street Liverpool, L3 2ET, Liverpool, UK; 2 University of Liverpool, Liverpool, UK; 3 Spital STS, AG Thun, Switzerland, Bournemouth University, Bournemouth, UK; 4 Oberhausen-Berg, Germany

**Keywords:** Conscience, Conscientious objection, Midwives, Nurses, Abortion, Systematic review

## Abstract

**Background:**

Freedom of conscience is a core element of human rights respected by most European countries. It allows abortion through the inclusion of a conscience clause, which permits opting out of providing such services. However, the grounds for invoking conscientious objection lack clarity. Our aim in this paper is to take a step in this direction by carrying out a systematic review of reasons by midwives and nurses for declining, on conscience grounds, to participate in abortion.

**Method:**

We conducted a systematic review of ethical arguments asking, “What reasons have been reported in the argument based literature for or against conscientious objection to abortion provision by nurses or midwives?” We particularly wanted to identify any discussion of the responsibilities of midwives and nurses in this area. Search terms were conscientious objection and abortion or termination and nurse or midwife or midwives or physicians or doctors or medics within the dates 2000–2016 on: HEIN legal, Medline, CINAHL, Psychinfo, Academic Search Complete, Web of Science including publications in English, German and Dutch. Final articles were subjected to a rigorous analysis, coding and classifying each line into reason mentions, narrow and broad reasons for or against conscientious objection.

**Results:**

Of an initial 1085 articles, 10 were included. We identified 23 broad reasons, containing 116narrow reasons and 269 reason mentions. Eighty one (81) narrow reasons argued in favour of and 35 against conscientious objection. Using predetermined categories of moral, practical, religious or legal reasons, “moral reasons” contained the largest number of narrow reasons (*n* =  58). The reasons and their associated mentions in this category outnumber those in the sum of the other three categories.

**Conclusions:**

We identified no absolute argument either for or against conscientious objection by midwives or nurses. An invisibility of midwives and nurses exists in the whole debate concerning conscientious objection reflecting a gap between literature and practice, as it is they whom WHO recommend as providers of this service. While the arguments in the literature emphasize the need for provision of conscientious objection, a balanced debate is necessary in this field, which includes all relevant health professionals.

## Background

Freedom of conscience is a core element of human rights that in Europe is protected in documents such as the European Convention on Human Rights (EHCR) [1]. Under this Resolution, any person who feels that his or her rights under the Resolution have been violated, has recourse to an appeal to the European Court of Human Rights whose judgements are binding upon the countries concerned. Article 9 of the Resolution specifically states that:

1. Everyone has the right to freedom of thought, conscience and religion; this right includes freedom to change his religion or belief, and freedom, either alone or in community with others and in public or private, to manifest his religion or belief, in worship, teaching, practice and observance.
2. Freedom to manifest one’s religion or beliefs shall be subject only to such limitations as are prescribed by law and are necessary in a democratic society in the interests of public safety, for the protection of public order, health or morals, or the protection of the rights and freedoms of others.

Around the same time as key stakeholders in Europe were discussing respect for conscience, another debate was being brought to the fore in health care and in political circles in some countries by women’s and social health lobbyists and commentators. Concerns had been raised that high numbers of women were dying or being seriously mutilated from illegal abortions [2]. Subsequently, laws ensuring the safe provision of abortion were gradually enacted throughout Europe and elsewhere, with abortion on request currently available in 69% of the world’s developed countries [3].

As such laws became enacted in each country, for the most part, they included a conscience clause which permitted opting out of providing such services on conscience grounds [4]. The 1967 UK Abortion Act, for example, states that “no one is under any duty to participate, contrary to his or her conscience, in any treatment authorised by the Act”; although the exemption does not apply where treatment “is necessary to save the life or to prevent grave permanent injury to the physical or mental health of a pregnant woman” [5]. In Europe these laws and the Convention on Human Rights have been supported and reinforced by the Council’s Resolution 1763, which specifically focuses on the right to conscientious objection in lawful medical care but ensuring that women’s lawful access to abortion is also protected [4].

Since the beginning of the twenty-first century the procedures for carrying out abortion have changed. Now, the widespread availability of the “morning after pill”, which can be prescribed by pharmacists, enables self-medication within 48 h of unprotected sexual intercourse. Similarly, in the early stages of pregnancy the woman may self-administer medication, under the prescription and supervision of a medical practitioner in approved premises. After about the 14th week of pregnancy, the procedure involves induction of labour and it is often midwives who are the main care providers during labour. These changes are strongly supported by the World Health Organisation (WHO) which recommends that midwives or nurses should be the key providers of abortion services [6]. Hence, the direct provision of abortion services concerns health professionals, such as midwives as well as nurses and doctors.

Abortion remains a morally contentious issue with some midwives and nurses declining to participate in it on the grounds of conscience. The International Confederation of Midwives’ Code of Ethics clearly supports this stance, stating that “midwives may decide not to participate in activities for which they hold deep moral opposition” [7] although this does not address how the necessary care may be provided. Conversely, the WHO’s recent guidelines on abortion [8] do not address the issue of conscientious objection at all.

When conscientious objection is discussed in relation to health care settings responsible for the provision of legal abortion, it often becomes the centre of acrimonious debate between practitioners as well as academics. They are divided as to the rights and wrongs both of the procedure itself and of health professionals’ objections to participating in the procedure. Pellegrino summed up the arguments as both the patient and the health professional being “entitled to respect for their personal autonomy. Neither one is empowered to override the other. The protection of freedom of conscience is owed to both” [9:241].

There is thus still polarisation on how much weight to give to the rights and responsibilities of health professionals to offer an abortion service and their rights to conscientiously object to participating in it. Authors on both sides of the debate state that European countries should critically assess the laws governing conscientious objection and their effects on women’s legal rights to a service [9, 10]. However, in this paper we argue that before such a step is taken, the grounds for invoking conscientious objection need to be clear. Our aim in this paper is to take a step in this direction by carrying out a systematic review of reasons by midwives and nurses for declining, on conscience grounds, to participate in abortion.

## Method

The idea of conducting systematic reviews of ethical arguments is a relatively new phenomenon. McCullough et al. argued that clinicians and policy makers would benefit from some kind of synthesis of the ethical arguments surrounding particular issues, and developed a methodology for conducting such reviews, which assessed quality-weighted conclusions reached in the ethical literature. This begins by formulating an ethical question and then assesses the conclusions of articles addressing that question [11]. Strech and Sofaer put forward a critique of this method arguing that McCullough et al.’s way of measuring the quality of the ethical arguments is inadequate, and that such reviews may mislead clinicians and policy makers. In the present review, we have used the method developed by Strech and Sofaer [12]. They strongly recommend that the question to be investigated should be factual rather than ethical, as in McCullough et al.’s approach. This was a key reason for our choosing this approach, as our focus was on evaluating the “reasons” put forward in the literature for or against conscientious objection by midwives and nurses to the provision of abortion services. Strech and Sofaer argue that their model aims to “improve ethically relevant decisions in healthcare, research or policy” which corresponds with our aim in this systematic review of reasons [13:125]. They propose a four step approach: formulating and reviewing both the question and eligibility criteria, identifying all the literature that meets the criteria, extracting and synthesising data, deriving and presenting answers to the review question.

### Formulation of the review question and eligibility criteria

The initial question we chose for this review was: “What reasons have been reported in the argument based academic literature for or against conscientious objection to abortion provision by health professionals?” Prior to beginning the review, to reflect the change in process of inducing abortion, we replaced “health professionals” with “midwives” or “nurses”. The modification was made as we had observed that the roles and perspectives of midwives and nurses often differed from other health professionals, such as pharmacists or physicians in these situations [13].

In order to answer the question we first agreed search terms of conscientious objection and abortion or termination and nurse or midwife or midwives or physician or doctor or medic (physician hereafter). We decided to include physicians in the review, as midwives and nurses are held by the World Health Organisation to be mid-level health care providers, subservient to physicians in most health service structures. If we had excluded them, then important literature may have been missed as often midwives and nurses appear in the text of articles but are not shown in the results of the search.

### Identification of all the literature meeting the eligibility criteria

We undertook an initial search within the dates 2000–2016 using the following databases: HEIN legal, Medline, CINAHL, Psychinfo, Academic Search Complete Web of Science including publications in English, German and Dutch. The dates reflect changes to abortion provision which have taken place at a faster pace in the twenty-first century than previously. Each of the authors took responsibility for one domain i.e. legal, ethical and health. In total 1085 records were identified; 1082 records through electronic database searches and a further 3 were later added from references.

In accordance with the chosen method, in the first screening round we checked the results of the search in each domain for suitability of inclusion in determining whether or not abortion was the primary focus of the article based on its title and abstract and excluded 792 articles. A total of 293 records were saved for further scrutiny. In the second round of screening we compared remaining articles from each database and removed a further 157 articles because of duplication.

**Fig. 1:**
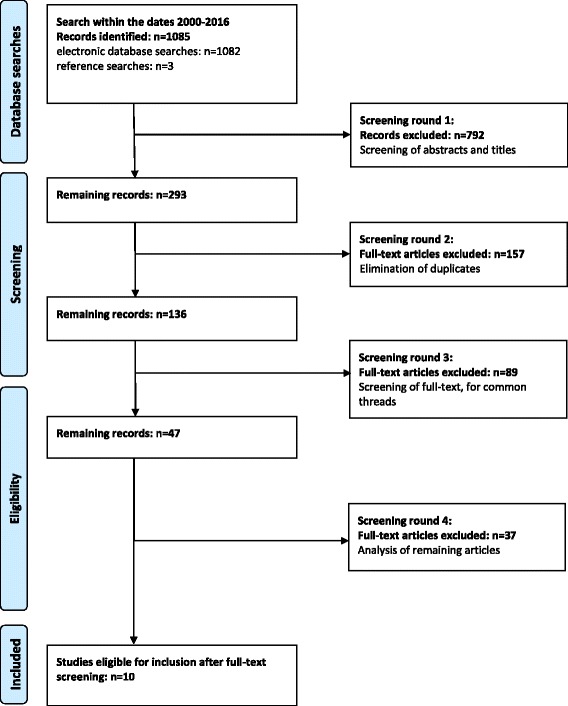
Electronic search for literature identification and the selection process

The full texts of the resulting 136 articles were screened in round three and each read by two authors looking for the common threads. They were checked with a focus on eligibility criteria (see below). At this stage, the team made the decision to eliminate those which only referred to physicians as they hold a more autonomous role than other health professionals in most health care systems This resulted in the exclusion of 89 articles. Forty seven articles remained and were screened in round four. Two of the authors read each independently and those which did not include clear reasons for or against conscientious objection were removed on this ground. The first two reviewers’ independent results tallied in all but three of the articles. The third reviewer then had access to the spreadsheets and concurred, mediating where necessary to reach a solution agreed by all. The screening process in round four resulted in an exclusion of 37 articles. The remaining 10 articles were then included in this review. The process is summarised in Fig. 1. Table 1 provides a list of all articles that were included in our systematic review of reasons.

**Table 1:** Final articles used

Bowman MS, Schandevel CP. The harmony between professional conscience rights and patients´ right of access. Phoenix L Rev. 2012; 6:31–62. Cavanaugh TA. Professional conscientious objection in medicine with attention to referral. Philosophy. Ave Maria L Rev. 2010;9:1 Chavkin W, Leitman L, Polin K. Conscientious objection and refusal to provide reproductive healthcare: a White Paper examining prevalence, health consequences, and policy responses. Int J Gynecol Obstet.2013;123:S41–56. Fovargue S, Neal M. ‘In good conscience’: conscience-based exemptions and proper medical treatment. Med Law Rev.2015;23(2):221–241. Greenawalt K. Refusals of conscience: what are they and when should they be accommodated? Ave Maria L Rev. 2010;9(1):47–65. Greenawalt K. Religious toleration and claims of conscience. J L & Pol. 2013;23:91–128. Pellegrino ED. The physician’s conscience, conscience clause, and religious belief: a catholic perspective. Fordham Urban Law J. 2002;30:221–44. Sawicki NN. The hollow promise of freedom of conscience. Cardozo L Rev.2012;4:1389–1449. Sepper, E. Taking conscience seriously. Va L Rev. 2012;98:1502–7155. Sepper E. Not only the doctor’s dilemma: the complexity of conscience in medicine. Faulkner L Rev. 2013;4:385–410.	

### Extraction and synthesis of data

Strech and Sofaer’s approach [12] recognises that interpretative work occurs when reasons are identified. They argue that the process of systematically reviewing reasons incorporates a detailed focus on the whole text and in different paragraphs that result in specific findings. Grounded in this understanding, they use the term “reason mention” for a reason expressed by a specific passage in the original text. We thus adopted the term “reason mention” in our study for all identified text passages that addressed a reason for or against conscientious objection to be faithful to the method.

Based on the identified reason mentions, Strech and Sofaer recommend the development of a hierarchy of narrow and broad reasons. This means that each reason mention becomes assigned first to a narrow and then a broad reason and ultimately a category.

According to Strech and Sofaer “narrow reason” is the term that is provided by the researcher for the previously identified reason mention. Each reason mention is initially attributed to a narrow reason after the first interpretative work of the researcher. We conducted this step of creating narrow reasons with the utmost care because our interpretative work was inevitably of a subjective nature. We ensured transparency by working with spreadsheets that allowed retracing all narrow reasons back to the original reason mention. The second loop of interpretative work according to Strech and Sofaer is to attribute each narrow reason to a “broad reason”. Broad reasons are the findings that emerge through the detailed process of systematically reviewing, interpreting and grouping narrow reasons together. Finally, Strech and Sofaer developed, at a higher level, categories encompassing all the broad reasons.

One of the authors first went through each article in detail and identified reason mentions in each of the selected publications. Spreadsheets were created for each reason mention and, using the qualitative software analysis tool MAXQDA, the same author grouped them into a number of narrow reasons. Thereafter, these narrow reasons were discussed with another author. Some of the reason mentions that we identified initially were discarded as they did not provide reasons for or against conscientious objection. We identified some arguments in the articles that were not directly related to our research question but were consequences that resulted from the arguments for or against conscientious objection. We summed them up under “consequence arguments” and did not discuss them further. The narrow reasons were then grouped together into broad reasons by the same two authors. Finally, we developed four categories, which encompassed all the reasons covered in the articles: legal, moral, practical and religious reasons. As also noted by Strech and Sofaer, some reason mentions could embrace more than one narrow reason, likewise some narrow reasons could embrace more than one broad reason and some broad reason could embrace more than one category. We addressed this initially where appropriate. However, ultimately, we decided in cases of such conflicts to place each narrow reason in only one broad reason and each broad reason in one category by joint decisions in order to avoid unnecessary confusion. The remaining two authors reviewed the final tables showing the categories, the broad and narrow reasons and the reason mentions.

### Derive and present results

Sofaer and Strech adopt a numerical hierarchy to prioritising results and in another of their publications they class the most frequently mentioned reason as the strongest [14]. The hierarchy for ordering them was the number of broad reasons, thereafter the number of narrow reasons and finally the number of reason mentions. We have followed this in the present article so as to remain faithful to the chosen method.

## Results

Of the 10 articles that we included, seven derive from legal scholars in the USA. Two come from physicians and one from a philosopher also based in the USA, the final being written by two lawyers in the UK. None were written by midwives or nurses.

Our review illustrated that there are more reasons that argue for the provision of conscientious objection than against it. Of the 116 narrow reasons we identified, 81 narrow reasons were for conscientious objection (70%) and 35 narrow reasons against conscientious objection (30%).

Table 2 shows the broad reasons, narrow reasons and reason mentions within the articles included in this review related to their associated categories of moral, practical, religious or legal reasons. It is immediately evident that within the four pre-determined categories, that of “moral reasons” had by far the largest number of both broad and narrow reasons and reason mentions. The reasons and their associated mentions in this category outnumber those in the sum of the other three categories.

**Table 2:** Overview of findings

	Number of broad reasons	Number of narrow reasons (for/against CO)	Number of reason mentions
Moral reasons	11	58 (47/11)	150
Practical reasons	5	30 (14/16)	52
Religious reasons	4	15 (12/3)	28
Legal reasons	3	13 (8/5)	39
Total	23	116 (81/35)	269

The detailed analysis of our findings identified broad and narrow reasons in every category. Tables 3, 4, 5, 6 present each of the categories with their related broad and narrow reasons for and against conscientious objection. Broad reasons are used as subheadings, each being followed by a bracket. The numbers used in this bracket refer to the number of narrow reasons that were used to argue for conscientious objection (+), the number of narrow reasons that were used to argue against conscientious objection(−) and the number of reason mentions (rm) that the broad reason contains. This means that, for example, the broad reason “respecting importance of conscience” (+ 11/− 1, rm.:24) includes 11 narrow reasons that were used to argue for and one narrow reason against conscientious objection, based on 24 reason mentions. The detailed analysis of each category separately was shown to be especially important for the category of moral reasons. This category showed that more than 80% of the total narrow reasons in all categories argued for the provision of conscientious objection. The predominant provision of narrow reasons in favour of conscientious objection was also identified in the categories of religious and legal reasons. However, the category of practical reasons showed slightly more narrow reasons that predominantly argued against the provision of conscientious objection.

**Table 3:** Moral Reasons

Moral Reasons	
Respecting importance of conscience or CO (+ 11/− 1; rm.:24)	
+	Conscience is an inner voice that requires to be listened to (6)
+	Freedom of conscience is a moral right (4)
+	CO reflects objective moral truth (2)
+	Conflicts of conscience are a regular feature of moral life (2)
+	Degree of intensity and magnitude of an act underlies moral judgment (2)
+	Society and Values, individual liberty and autonomy (1)
+	Respects personal beliefs (1)
-	Moral judgment leads to` values´ (2)
+	Conscience is the “law of the intellect” (1)
+	Conscience is related to personal identification (1)
+	Conscience implies a strong moral conviction (1)
+	Conscience involves a certain intensity of conviction (1)
Criteria for CO (+ 9/−2; rm.:36)	
-	Physicians must separate moral belief from professional life (9)
+	HCP’s position must be consistent with their other beliefs and actions (7)
+	Conscience is not one-sided (7)
+	HCP have differing attitudes towards conscience (2)
+	Objection must be to the treatment (2)
+	Position held must be sincere (2)
+	Rationale must reflect valid view of service’s goals (2)
+	Position emerges after alternatives considered (2)
+	Position must fit within coherent system of ethical beliefs (1)
+	Mystifying characteristic: Not backed by reason or logic (1)
-	Position poses risk to HCP’s moral integrity (1)
Moral integrity needs to be respected (+ 5/−1; rm.:22)	
-	Women have an access right (12)
+	Acting against own conscience causes moral distress (4)
+	Patient has a moral right to informed consent and refusal (3)
+	Conscience may extend beyond moral reasoning (1)
+	Most compelling moral basis (1)
+	Protection of individual liberty (1)
Normative value of CO (+ 4/−1; rm.:13)	
+	Fundamental principle of a pluralistic society (4)
+	Core of humanity (3)
+	Intrinsic value is autonomy and human flourishing (3)
+	Conscience is not infallible (2)
-	CO as synonym for refusal to deliver abortions (1)
CO protects HCP (+ 5/−0; rm.:9)	
+	Conscientious position is an “ethical position” of a HCP (5)
+	CO credits the individual conviction against general perception (1)
+	CO encompasses more than simply not performing the intervention (1)
+	CO considers one’s own conduct not that of another (1)
+	CO is a vehicle for HCP who regard such requests objectionable (1)
Conscience is closely related to identity and sense of self (+ 4/−1; rm.:8)	
+	Conscience is central to being a whole person (2)
+	Conscience is experienced in relation to own actions (2)
-	Toleration of moral diversity is plausible and questionable (2)
+	Conscience is a driver of human behaviour (1)
+	Failure to follow own conscience generates regret and guilt (1)
Respect for autonomy (+ 3/−1; rm.:10)	
-	CO may heighten risk for women living in precarious circumstances (5)
+	Value-neutral care is impossible to be provided (3)
+	Professional CO reflects autonomy of the profession (1)
+	Choices of the patient may be ethically unacceptable (1)
Ignoring conscience of HCP is a form of discrimination (+ 3/− 0; rm.:3)	
+	Loss of self-respect (1)
+	Discrimination against well-performing practitioners (1)
+	Discrimination related to religious conscience (1)
Requirement to offer a service (+ 1/−2; rm.:11)	
-	Three main arguments for “access” can be rebutted (4)
-	There are biased assumptions of forced access position (4)
+	Consequences follow when conscience rights are eliminated (3)
Freedom of conscience (+ 2/−0; rm.:7)	
+	Both the “willing” / “refusing” provider have conscience (6)
+	Conscience is a societal value (1)
Imposing own beliefs (+ 0/−2; rm.:7)	
-	Violation of physician’s conscience (5)
-	Patient care over adherence to religious doctrines or self-interest (2)

**Table 4:** Practical Reasons

Practical Reasons	
Institutional refusal (+ 4/− 10; rm.:23)	
-	Greater risk of patient injury in emergencies (3)
-	Patients have fewer options (3)
-	Fear that CO becomes widespread (2)
-	Encourages refusal unrelated to moral reasoning (2)
-	CO as exemption from general duties to obey the law (2)
+	CO cannot be limited to individuals (2)
+	Ethical and religious directives for Catholic health care (2)
+	May help HCP to change initial view (1)
+	Undervaluation of moral associations (1)
-	Limits patient access (1)
-	Failure of dissenting staff for emergencies (1)
-	Best practice may not be possible for the HCP (1)
-	Right to refuse may end in right to dictate care (1)
-	Conflicts between CO and medical technologies (1)
Justifying professional CO (+ 3/−3; rm.:9)	
-	No common sense of what is “wrong” causes no need for provision (3)
+	CO is evidence-based (2)
-	HCP with strong CO is torn between belief and requirement (1)
-	Formalistic argument to provide no exemption officials (1)
+	Institutions can be selective in offering services (1)
+	HCP may lack the intellectual or verbal skill to express CO (1)
Practice of disclosure creates risk for the HCP (+ 5/−0; rm.:13)	
+	Professional disadvantages (7)
+	Suffers embarrassment and inconvenience (2)
+	Vulnerable to attacks from the other side (2)
+	Disadvantages in asserting claims (1)
+	Experiences personal safety in danger (1)
Degree of involvement among HCP is different (+ 1/− 2; rm.:5)	
+	Expectations change over time (2)
-	Intrinsic relevance is debatable (2)
-	Function in a job is straightforward (1)
Organisational ethics require consideration (+ 1/−1; rm.:2)	
-	Choices constrained in emergencies when the closest hospital is far off (1)
+	Benefit for society (1)

**Table 5:** Religious Reasons

Religious Reasons	
Religion does not permit involvement (+ 6/−1; rm.:16)	
+	Religious law should be followed (6)
+	It is a sin against God (2)
+	Religious premises are true (2)
+	Job was chosen due to `calling´ (2)
+	Conscientious objection due to eternal welfare (2)
+	Nonreligious-based argumentation of CO reflects professional position (1)
-	Catholic physicians should not become gynaecologists (1)
Religious convictions form conscience (+ 3/−0; rm.:7)	
+	Humans have values (3)
+	Religious based reasoning against the provision of abortion related care (3)
+	Religious HCP’s are confronted with serious conscience conflicts (1)
Religious toleration has multiple dimensions (+ 1/−2; rm.:3)	
+	Patient’s religious values can collide with medical providers (1)
-	Recognising an exemption is not necessarily basis for opposition (1)
-	Conflicting claims of toleration and how to respond (1)
Controversies in religion-based argumentation (+ 2/−0; rm.:2)	
+	Intrinsic plausibility of religious conclusions is difficult (1)
+	Conscience is equated with natural law (1)

**Table 6:** Legal Reasons

Legal Reasons	
Safeguarding conscience (+ 6/−1; rm.:23)	
+	Ethical dilemma can be addressed (7)
+	Protects HCP’s conscience (4)
+	Acknowledges legitimacy of conscience at institutional level (4)
+	Protection for individuals’ consciences (3)
-	Authorises discriminatory refusals (2)
+	Should encourage ethical deliberation (2)
+	Provides a basis for professional grounds of CO (1)
Legality argument (+ 2/−2; rm.:14)	
-	HCP must suspend prolife conscience (5)
-	Requires professionals who make it available (5)
+	Codified conscience laws are just as legal as abortion (3)
+	Legality is not a binary concept (1)
Critique of the conscience clause (+ 0/−2; rm.:2)	
-	Licenses harm to patients (3)
-	Overlooks patient’s moral integrity (3)

In line with our search for reasons of argument-based literature addressing conscientious objection, we not only analysed each category, as outlined in Tables 3, 4, 5, 6, but also considered each broad reason in detail independently of its attributed category. This “trans-categorical view” enabled us to focus on the content and on the argument itself. Table 7 provides an overview of all identified broad reasons that were used to argue for or against conscientious objection in the literature included in this review. It also includes one example of a reason mention underpinning each broad reason.

**Table 7:** Overview of broad reasons used to argue for or against CO

Broad reasons used to argue for or against CO		Example of reason mentions
++: Broad reason containing only narrow reasons arguing for CO	CO protects HCP (+ 5/− 0; rm.:9)[MR]	“We live in a society that has become increasingly individual over time, with citizens encouraged to seek what is best for themselves. In one sense, a right of conscience is a counter, focusing as it does on perceived obligation, not self-satisfaction. But the right is strongly individualistic, crediting the individual’s conviction against the general perception of what is socially desirable. One might think that creating a legal right, especially a broad one not limited to religious conviction, will contribute to an unhealthy sense that each individual judges for herself, giving little or no weight to a sense of community and to prevailing opinions within the society about what is needed.”[15]
	Ignoring conscience of HCP is a form of discrimination (+ 3/− 0; rm.:3) [MR]	“Their feeling that they have yielded to compulsion and violated their most deeply held beliefs and principles may involve profound resentment and loss of self-respect.” [18]
	Freedom of conscience (+ 2/− 0; rm.:7) [MR]	“They assert that, because provision of care can be conscience based, full respect for conscience requires accommodation of both objection to participation and commitment to performance of services such that the latter group of providers also have the right to not suffer discrimination on thebasis of their convictions.” [19]
	Practice of disclosure creates risk for the HCP (+ 5/− 0; rm.:13) [PR]	“Ironically, in most jurisdictions, the same facility-religious or not-may alternate between refusing and willing. For example, a clinic that only refuses to provide nontherapeutic abortions typically will have to accommodate a doctor who will not participate in therapeutic abortions, sterilizations, or contraceptive care.” [21]
	Religious convictions form conscience (+ 3/− 0; rm.:7) [RR]	“Religious beliefs, which statutes and philosophical traditions recognize as a basis for acts of conscience, may be of as fundamental significance to a willing provider as they are to a refuser.” [21]
	Controversies in religion-based argumentation (+ 2/− 0; rm.:2) [RR]	“To highlight exclusively religiously based conscientious objection to the neglect of professional conscientious objection renders conscientious objection a strange and alien phenomenon to the nonreligious. More importantly, to do so erroneously suggests that the professional has no positions concerning the ethics of her own practice.” [20]
+: Broad reason containing predominantly narrow reasons for CO	Respecting importance of conscience or CO (+ 11/− 1; rm.:24) [MR]	“When we describe a person as having acted on the grounds of conscience, we typically mean that she “acted on the basis of a sincere conviction about what is morally required or forbidden.”15 Thus, claims of conscience can be understood as a subset of moral claims generally one that connotes a strong link with individual identity and a preference for suffering significant burdens rather than acting against conscientious belief.” [18]
	Criteria for CO (+ 9/− 2; rm.:36) [MR]	“It must be consistent with the HCP’s other beliefs and actions, particularly those in proximate areas of concern.” [24]
	Moral integrity needs to be respected (+ 5/− 1; rm.:22) [MR]	“A moral system that tolerated intolerance would seem internally inconsistent’.” [24]
	Normative value of CO (+ 4/− 1; rm.:13) [MR]	“Conscience, however, is not so one-sided. Nor is medical decision-making so straightforward. First, medical decisions -especially those involving questions of life and death - inspire divergent moral convictions. Second, as I will explain, medical decisions do not simply implicate conscience for the provider. They should be thought of instead as involving, at minimum, three parties: patients, providers, and institutions. This three-sided relationship complicates moral decision-making, with each party asserting potentially conflicting claims.” [22]
	Conscience is closely related to identity and sense of self (+ 4/− 1; rm.:8) [MR]	“Acting according to conscience has real importance less because it is about being (morally or politically) right than because it is central to being a whole person. Both theory and experience indicate that conscience is closely related to one’s moral integrity or sense of self.” [21]
	Respect for autonomy (+ 3/− 1; rm.:10) [MR]	“Professional conscientious objection in medicine is an instance of the autonomy of the professions from what is simply legal.’ Professional conscientious objection differs from religiously grounded objection by being reason-based.” [20]
	Religion does not permit involvement (+ 6/− 1; rm.:16) [RR]	“A different basis for possible differentiation concerns what is at stake. Perhaps religious objectors usually perceive that more is at stake, including their eternal welfare. This sense of magnitude of impairment might be related to what a claimant would be willing to sacrifice to avoid doing a wrongful act.” [15]
	Safeguarding conscience (+ 6/− 1; rm.:23) [LR]	“As a two-way street, the conscience clause acknowledges the legitimacy of conscience at the level of institutions, while preventing institutions and individuals from discriminating against those whose consciences differ.” [20]
+−: Broad reason with equal amount of narrow reasons for and against CO	Justifying professional CO (+ 3/− 3; rm.:9) [PR]	“A final variation concerns public attitudes. If the community is deeply divided over whether a form of health care involves a serious wrong, there is a powerful argument that no individual or institution should be required to provide it.” [15]
	Organisational ethics require consideration (+ 1/− 1; rm.:2) [PR]	“Organizational ethics is a systematic examination of the morality of collective actions in human institutions dedicated to some specific purposes in society. The ethical “code” or commitment of a specific institution is now customarily expressed in its mission statement. This is in a way the “conscience” of the institution.” [17]
	Legality argument (+ 2/− 2; rm.:14) [LR]	“Ultimately, there is no real possibility of engaging in the conscience rights discussion with total deference to the law because the discussion is precisely about what the law should be. In the end, the legality argument is tautological and fails to advance the claims made by forced-access advocates.”[23]
-: Broad reason containing predominantly narrow reasons against CO	Requirement to offer a service (+ 1/− 2; rm.:11) [MR]	Already we hear ethicists suggesting that physicians must separate their personal moral beliefs from their professional lives if they wish to practice in a secular society and remain licensed as fully functioning physicians. [17]
	Institutional refusal (+ 4/− 10; rm.:23) [PR]	“When an entire institution refuses to deliver common medical procedures, like contraception and abortion, the risk to patients is further magnified. First of all, access becomes a more significant issue. Patients’ choice of a healthcare facility is more limited than their choice of an individual doctor.” [22]
	Degree of involvement among HCP is different (+ 1/− 2; rm.:5)[PR]	“The intrinsic relevance of degree of involvement is more debatable. According to most people’s ordinary sense, if a person’s job calls upon her to receive answers from questionnaires that admitted patients have answered and to exchange a few words with those patients, an objection to such contact with the patients who happen to be entering to receive abortions would be unreasonable.” [15]
	Religious toleration has multiple dimensions (+ 1/− 2; rm.:3) [RR]	“Moreover, secular religiousity, which supposedly tolerates differences, does so only within a narrow range of so-called “values” that are supposedly “free” of religious or religious taint. But secular religiousity is itself an orthodoxy. Its “values” are based in democratic procedures, personal preference as the basis for religious choice, commitment to a free market economy, the commodification of health care, and an eschewal of religious belief. To deviate from this notion of religious “neutrality” in public policy is to be “undemocratic,” prejudiced, and intolerably sectarian.” [17]
- -: Broad reason containing only narrow reasons against CO	Imposing own beliefs (+ 0/− 2; rm.:7) [MR]	“The “Imposing Your Beliefs” Argument Imposes a Rejection of Hippocratic Principles.” [23]
	Critique of the conscience clause (+ 0/− 2; rm.:2) [LR]	“Immunity goes far beyond what is necessary to protect the moral integrity of medical providers. It destabilizes the medical profession’s duties to do no harm and respect patient autonomy. It endangers the very trust upon which the profession relies.” [22]

When we explored all the broad reasons in detail, we identified eight as containing narrow reasons that either argued only for or against conscientious objection. Six of these, embracing all four categories, supported conscientious objection while two, one from each of moral and legal reasons, were against it. Nine broad reasons contained narrow reasons predominantly arguing for conscientious objection while three were neutral and four were mainly against conscientious objection. Such figures show that there is no category in which the arguments were entirely in one direction. This is addressed next.

## Discussion

This systematic review of reasons has shown that the identified literature contains many reasons embracing a wide spectrum of arguments both for and against conscientious objection to the provision of abortion care by nurses or midwives. Most of the authors of the selected articles state their arguments in ways which leave the reader in no doubt as to their stance although Greenawalt [15, 16] attempts to remain neutral through posing a number of questions to stimulate readerss thoughts. The majority of the authors accept the need for some form of conscientious objection by nurses (and, by default, midwives) The other articles are clearly in favour of one side of the debate with two specifically concentrating on the Christian faith [17, 18]. Most articles seek clarifications about limits to which conscientious objection should be permitted, one suggesting that the idea of referral could be seen as “complicity in wrongdoing” [19:239] but the majority taking the approach that some form of referral is necessary [17, 19, 20]. Sepper’s two articles focus strongly in favour of presenting arguments for or against institutional “consciences” [21, 22].

The polarities found in our current review are articulated in one article’s finding that the existing debate about conscientious objection is characterised by conflicting positions between patients’ right to access legal health services and health professionals’ right to practise with respect to their own consciences [23]. Such conflicts are experienced by midwives and nurses who are confronted with the “dual commitment” noted in two of the publications [21, 22]. In order to illustrate this notion of “dual commitment” further, we now discuss two broad reasons that differed in their line of argument but containing similar numbers of reason mentions. The broad reason “respecting importance of conscience” is an exemplar of an argument predominantly in favour of conscientious objection. It contains 11 narrow reasons for and one against conscientious objection with a total of 24 reason mentions. We then selected “institutional refusal” as an example of a broad reason that was used to argue predominantly against individual freedom of conscientious objection with 10 narrow reasons against conscientious objection and four in its favour with 23 reason mentions. By making this selection, rather than offering a detailed discussion of the broad reasons arguing only in one direction, we acknowledge and support the claim for a content-neutral approach to the accommodation of conscience as recommended by Sawicki who stated that respecting various available arguments is an important principle for a pluralistic society [18]. The discussion of these two broad reasons also notes Sepper’s warning to take conscience seriously by reflecting upon all available arguments [21]. These two selected reasons will now be discussed in relation to the literature we analysed.

As noted previously, the broad reason: “Respecting importance of conscience”, that was derived from five of the 11 selected articles, was predominantly in favour of conscientious objection by nurses or midwives. Sawicki, arguing particularly powerfully that conscience exists within all human beings from the beginning of life, suggested that “even the youngest child has some understanding of right and wrong as the nagging feeling of shame when she has made the wrong decision” suggests. She further argued that at times a person’s conscience is so powerful that it compels that person to act against the dominant trends and feeling bound by “volitional necessity” [18:1395]. Pellegrino supports this argument claiming that conscience impels a person to act in a certain way, “the judgments of conscience are morally binding, i.e., they must be followed or the moral agent has acted immorally and accountably” [9:227]. Like Sawicki [18], he goes further, suggesting that “to ignore this ‘inner voice’ is to induce guilt, remorse, and shame. Only the amoral sociopath escapes the grip of conscience” [9:227]. Greenawalt, similarly, referred to the “inner voice” by arguing from the stance of the “intrinsic nature of conscience” and suggesting that if people felt strongly that “an act would violate their conscience, they will be hesitant to perform the act, even under pressure” [17:97]. Sepper, however, argued from a different perspective, proposing that conscience “may be experienced retrospectively, generating guilt or regret, or prospectively, generating a sense that failure to resolve these conflicting demands will risk one’s sense of self,” thereby coming to the fore following the actions rather than preceding them [22:1528]. While all these authors therefore agreed that respecting conscience was important, there was failure to agree as to the nature of conscience and when it manifested itself.

The second broad reason we selected was “institutional refusal” which was derived from the arguments of six authors, four of whom also contributed to the reason discussed above. It respects that the decision to invoke a conscientious objection by a nurse or midwife to the provision of abortion services means that other health professionals may be required to assume an additional workload that they may resent. Two of the articles argued that allowing conscientious objection may lead to a greater risk of patient injury in emergencies, Sepper stating, “the presence of any refuser risks delays, the traumatizing of patients, and bodily harm” [22:1552}. Each of these authors also argued that allowing conscientious objection to be invoked gave patients fewer options in other areas of reproductive care, Sepper noting, “In the most difficult cases, a particular service might become unavailable in a particular community because the sole specialist…. refuses to provide it”. [22:1569]. However, the author does not suggest what these services might be and how this cause and effect would come about. Chavkin et al., whose article took an international perspective, supported this line of reasoning providing a visual schema outlining potential ramifications of refusal such as increased maternal mortality and morbidity and stigmatising of providers who are prepared to provide the service. However, as these authors have cautioned, this was derived from a process of logical reasoning rather than from the collection of empirical data [14].

In addressing this issue, Fovargue and Neal suggested that there was a need to take a step back as a, “lack of clarity about the proper limits of conscientious refusal to participate in particular healthcare practices has given rise to fears that, in the absence of clear parameters, conscience-based exemptions by midwives may become increasingly widespread, leading to intolerable burdens on health professionals, patients, and institutions” [19:221]. Sepper summed up both sides of the argument suggesting that institutional refusal may actually encourage refusal unrelated to moral reasoning and that conscience legislation “is under-inclusive with regard to willing providers and patients alike… At the same time, the legislation is over-inclusive, sweeping in refusal for reasons unrelated to conscience. So doing, it short-circuits ethical discussion and development” [23:403].

Arguing in favour of conscientious objection, Cavanaugh [20] proposed that conscientious objection cannot be limited to individuals as “to exclude institutional health providers from conscience clause protection is merely an indirect way of denying the conscience and morality of the individuals whose will and purposes the entities were created to effect” [21:196]. Using the example of institutions run by the Catholic Church, Sepper noted that ethical and religious directives for Catholic health care exist in the United States and as a condition of employment all medical providers [including midwives and nurses] are required to follow the Ethical and Religious Directives for Catholic Health Care Services. She suggests that by denying institutional conscientious objection, “this proposal effectively prevents those facilities that actually bring together people based on shared convictions from forming associations and excluding dissenters”, thereby denying the rights of individuals [22:1560]. Pellegrino concurs, arguing “the right to refuse care has rapidly metamorphosed into a right to demand and dictate the details of care” [9:223].

This more detailed look at the two selected broad reasons illustrates some of the diversity in not only the selected literature but also in the many articles that were considered for inclusion in this review. It further highlights how the same arguments can be used to illustrate both arguments for and against conscientious objection by nurses, midwives and other health care providers. As the number of narrow reasons contained within each broad reason become more balanced, this point was emphasised. For example, in the category of “practical reasons”, the broad reason “justifying professional conscientious objection” contained three narrow reasons in favour and three against conscientious objection, Sawicki summing it up as “because individuals with strong conscientious beliefs may find themselves torn between their beliefs and the requirements or expectations of the law” [18:1400]. She is countered by Chavkin et al. who state unequivocally that “providers have a professional duty to follow scientifically and professionally determined definitions of reproductive health services, and not to misrepresent them on the basis of personal beliefs” [20:550]. With a reference to our findings, it seems unlikely that middle ground will ever be found between these two views and generally in this whole debate.

### Limitations

While our review applied Strech and Sofaer’s method [12] we noted a number of limitations associated with this approach. Although a numerical consideration is the approach taken by Strech and Sofaer in evaluating argument-based literature, it lacks the in-depth consideration of important but rare arguments. While respecting the number of reason mentions that brought the two broad reasons “respecting importance of conscience” and “institutional refusal” to the top of our list, we agreed within our team that other broad reasons deserve attention even if mentioned less frequently. We also identified that there are specific narrow reasons that are worthy of attention despite being rarely mentioned, for example the narrow reason “conscience is the law of the intellect” in which the underlying reason mention states, “conscience is called “the law of our intellect” because it is a judgment of reason deduced from natural law” [9:226]. We consider this argument as important, because it views conscience as something special that interacts both with the intellect and the “law” intellectuals give themselves. However, it only attracted one reason mention. This example made us reflect upon the general nature of conducting a systematic review of reasons and conclude that understanding the broad reasons in our systematic review of reason may have benefitted from a more individual discussion of each broad reason apart from its numerical and hierarchical classification. Therefore, the contribution of our review is to bring into relief how people argue about accommodating conscientious objection and the reasons they use to support their arguments so that it can become clearer the grounds on which people are basing their arguments. By concentrating on the deliberative element of the debates, it may be possible to find areas of agreement between different positions, or at least areas where some common ground may be formed.

Despite the rigorous process we adopted for selecting the articles, it can clearly be seen that with one exception [24], all of them come from publications originating from the United States of America. Similarly, seven of the articles’ authors are lawyers, the remaining four authors being two physicians and two philosophers. It was disappointing that only one article selected came from Europe and none was written by nurses or midwives. This caused us to reconsider our final selection strategy in stage four when we reduced 47 articles to the final 10. One article in that round had been written by a nurse based in the UK but, although it contained information relevant to the change in techniques of inducing abortion, we rejected it because it was focused primarily on the management of conscientious objection rather than reasons for it [25].

All the articles chosen did discuss nurses, but midwives were scarcely mentioned possibly because midwives do not have a strong presence in the USA. The only article that specifically discussed midwives was that of Forvague and Neal [24] which came from Europe. Several authors suggested that while they primarily referred to physicians they also included nurses with Cavanaugh defining nurses and pharmacists as “medical professionals” while others such as ultrasound technicians and radiographers are not [21: 190]. We hypothesise that if more articles were written in Europe midwives would be more likely to be considered.

## Conclusion

The whole area of conscientious objection remains a very sensitive topic that needs to be considered in its entirety respecting both the arguments for and against its provision. We identified no arguments solely for or against conscientious objection by midwives or nurses. Our findings emphasise that, in order to address the issue of accommodating conscientious objection appropriately, multiple dimensions/perspectives have to be considered. The arguments in the literature predominantly emphasise the need for accommodating conscientious objection, therefore a balanced debate focusing on a variety of available arguments is necessary in this multi-faceted field of research. It is important that such debate considers the rights of midwives and nurses not to have to provide such a service if it is contrary to their conscience, and not only on the rights of women seeking abortions.

Further, we conclude that during the stage of identifying literature that meets the eligibility criteria for this review, midwives and nurses remain invisible, either hidden in the more generic “health” or even “medical” professionals, in the debates over conscientious objection. Additionally, few midwives and nurses have published in this area with no article authored by a nurse or midwife meeting our inclusion criteria. This reflects a gap between existing literature and practice because not only are midwives and nurses frequently confronted with providing abortion services in practice but the major policy drivers from WHO recommends them as the key providers of first and mid trimester abortions. The developing role of midwives and nurses in this area therefore merits urgent consideration and there is a need for both theoretical and empirical research to investigate the changes in abortion practice and how this affects the roles and responsibilities of midwives and nurses and how, in turn, this impacts on the conscientious objection debate.

## Abbreviations

CO: Conscientious objection
ICM: International Confederation of Midwives
RM: Reason mentions
WHO: World Health Organisation
